# Relation between systemic arteriosclerosis and choroidal blood flow in patients with acute coronary syndrome

**DOI:** 10.1038/s41598-025-25526-y

**Published:** 2025-11-24

**Authors:** Yasunari Ebuchi, Taiji Nagaoka, Kushiyama Akifumi, Daisuke Fukamachi, Riku Arai, Keisuke Kojima, Yuki Saito, Daisuke Kitano, Harumasa Yokota, Satoru Yamagami, Yasuo Okumura

**Affiliations:** 1 Division of Cardiology, Nihon University Itabashi Hospital, Tokyo, Japan; 2 Division of Ophthalmology, Nihon University Itabashi Hospital, Tokyo, Japan; 3 Department of Pharmacotherapy, Meiji Pharmaceutical University, Tokyo, Japan

**Keywords:** Acute coronary syndrome, Arteriosclerosis, Atherosclerosis, Brachial-ankle pulse-wave velocity, Left ventricular remodeling, Laser speckle flowgraphy, Ocular circulation, Cardiology, Pathogenesis

## Abstract

Blood flow parameters in the optic nerve head are associated with systemic atherosclerosis, early renal dysfunction, and diastolic cardiac dysfunction in patients with acute coronary syndrome (ACS). This study aimed to investigate the relationship between choroidal blood flow parameters evaluated using laser speckle flowgraphy (LSFG) and systemic atherosclerosis, cardiac function, and coronary artery disease severity in patients with acute coronary syndrome. We evaluated 44 patients admitted to the coronary care unit of Nihon University Itabashi Hospital for ACS between April 2019 and September 2020. Laser speckle flowgraphy was used to measure the mean blur rate (MBR) in three regions; the optic disc, and the choroidal region 1 disc diameter from the disc choroid temporally and nasally. Significant correlations were observed between the choroid MBR and systemic parameters in patients with ACS, including brachial-ankle pulse-wave velocity (*r* = 0.33, *P* = 0.029) and left ventricular diastolic diameter (*r* = − 0.30, *P* = 0.045). The choroidal MBR in the nasal region was significantly lower in the multivessel disease group than in the single vessel disease group (6.6 ± 1.6 vs. 8.5 ± 2.6, *P* = 0.006). The choroidal MBR was associated with systemic atherosclerosis, left ventricular remodeling, and coronary artery disease severity in patients with ACS, suggesting that it could be a useful non-invasive comprehensive arteriosclerotic marker.

## Introduction

Acute coronary syndrome (ACS) is associated with old age, hypertension, and type II diabetes mellitus and is strongly associated with systemic arteriosclerosis^1^. Transient retinopathy (soft exudate) occurs in patients with acute myocardial infarction (AMI) after percutaneous coronary intervention (PCI), suggesting that ACS may be involved in ocular circulation impairment^2,3^. Laser speckle flowgraphy (LSFG) can noninvasively detect impairments in ocular microcirculation by measuring blood flow velocity^4^. Using this technique, we have previously revealed that blood flow parameters in the optic nerve head are associated with systemic atherosclerosis, early renal dysfunction, and diastolic cardiac dysfunction in patients with ACS^5^.

Most of the studies on the choroid in patients with cardiovascular diseases assessed choroid thickness, and patients with coronary artery disease (CAD) were found to have thinner choroids than those of controls ^6,7^. Seo et al. reported that the choroidal vascularity index (CVI) was a significant factor associated with the presence of triple-vessel disease^8^. The results of those clinical trials suggest that choroidal circulation may be a good indicator of the severity of ACS.

Despite the high systemic atherosclerotic burden in patients with ACS, few studies have investigated whether systemic arteriosclerosis is associated with choroidal mean blur rate (MBR). LSFG enables the evaluation of choroidal blood flow by setting regions of interest at the parapapillary choroid to obtain the choroidal MBR^9^. This study aimed to investigate the association of systemic atherosclerotic parameters (brachial-ankle pulse wave velocity [baPWV]) and cardiac function assessed using transthoracic echocardiography with choroidal MBR evaluated using LSFG in patients with ACS. In addition, we also examine if choroidal MBR may be associated with multivessel disease (MVD), which is defined as left main coronary artery lesions or lesions involving two or more branches, because the presence of MVD is related to cardiac prognosis and treatment decisions^10^.

We intentionally selected patients in the acute phase of ACS for this study. This approach allowed us to measure the relationship between baseline systemic parameters and choroidal blood flow before long-term pharmacological therapies, such as intensive lipid-lowering or new antihypertensive drugs, could substantially alter the results, thereby reducing potential confounding factors.

## Results

### Patient characteristics

Table 1 summarizes the patient characteristics, choroidal blood flow results, and transthoracic echocardiographic parameters. Forty-four patients with a mean age of 65 ± 13 years were included in this study.

**Table 1:** Characteristic of the patients.

Baseline clinical data	*N* = 44
Age, years	65 ± 13
Male, n(%)	41 (93)
Systolic blood pressure, mmHg	115 ± 15
Diastolic blood pressure, mmHg	67 ± 10
Heart rate, beat per minutes	70 ± 8
HTN, n(%)	29 (66)
DM, n(%)	12 (27)
DLP, n(%)	27 (61)
CKD, n(%)	6 (14)
Smoking, n(%)	31 (70)
STEMI, n(%)	33 (75)
SYNTAX score	16 ± 7
Multivessel disease	22 (50)
Atrial fibrillation, n(%)	7 (16)
**Laboratory data**	
BUN, mg/dL	16 (12–19)
Creatinine, mg/dL	0.84 (0.71–0.96)
eGFR, ml/min/1.73 m^2	70.3 ± 20.5
LDL-Cho, mg/dL	115 ± 34
HDL-Cho, mg/dL	44 (38–50)
TG, mg/dL	131 (55–203)
HbA1c, %	6.0 (5.6–6.7)
NT-ProBNP, pg/mL	205 (57-2704)
**Medications**	
ACEI or ARB, n(%)	14 (32)
β-blocker, n(%)	6 (14)
Statin, n(%)	10 (23)
**Systemic atherosclerosis parameter**	
IMT, mm	2.3 (1.2–2.7)
ba-PWV, cm/s	1473 (1211–1885)
**Echocardiographic data**	
LVDd, mm	48 ± 6
LVDs, mm	33 ± 7
LVEF, %	57 ± 9
E, cm/sec	70 (56–87)
A, cm/sec	73 (61–87)
e’, cm/sec	6 ± 2
E/e’ ratio	11 (8–15)
LV mass index, g/m^2	91 ± 23
**Ophthalmic parameters**	
Soft exudate, n(%)	15 (34)
Intraocular pressure, mmHg	13 ± 3
Retinal MBR	20.3 ± 5.1
Choroidal MBR (Temporal)	5.5 ± 1.8
Choroidal MBR (Nasal)	7.6 ± 2.3

Values are the mean ± 2SD, median and interquartile range, or n(%) of patients.

A, peak mitral A wave velocity; ACEI, angiotensin converting enzyme inhibitor; ARB, angiotensin receptor blocker; baPWV, brachial-ankle pulse-wave velocity; BUN, blood urea nitrogen; CKD, chronic kidney disease; DM, diabetes mellitus; DLP, dyslipidemia; E peak mitral E wave velocity; e’ peak early diastolic myocardial velocity at septal position recorded by tissue Doppler imaging; E/e’ ratio, ratio of peak mitral E wave velocity to peak early diastolic myocardial velocity at the septal position by tissue Doppler imaging; eGFR, estimated glomerular filtration rate; HbA1c, hemoglobin A1c; HDL-Cho, high-density lipoprotein cholesterol; HTN, hypertension; IMT, intima media thickness; LDL-Cho, low-density lipoprotein cholesterol; LVEF, left ventricular ejection fraction; LVDd, left ventricular diastolic dimension; LVDs, left ventricular systolic dimension; LV mass index, left ventricular mass index; MBR, mean blur rate; NT-ProBNP, N-terminal pro-brain natriuretic peptide; STEMI, ST elevated myocardial infarction; TG, triglyceride.

*Obtained by Student *t* test, Mann-Whitney *U* test, Chi square test, or Fisher’s exact test, as appropriate.

### Choroidal MBR in relation to the presence or absence of multivessel disease

**Fig. 1:**
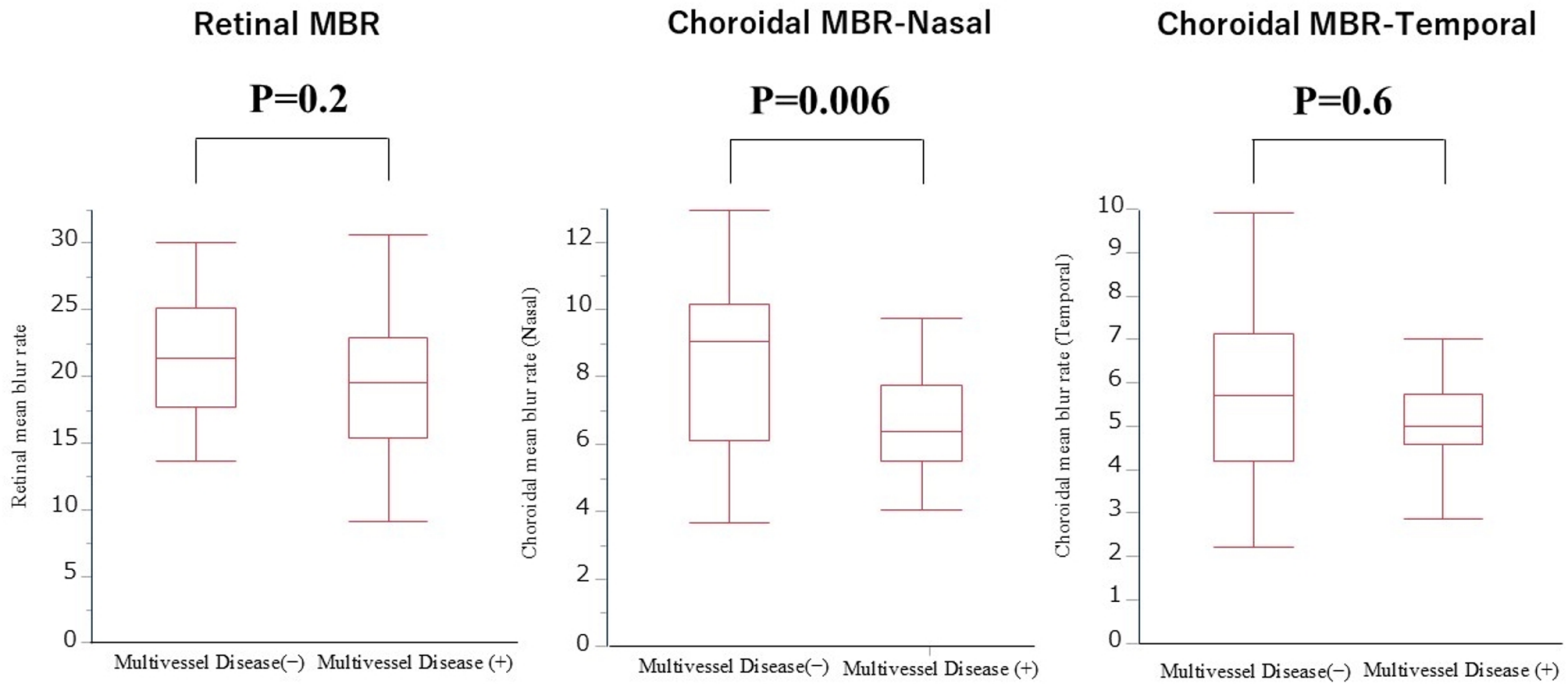
Choroidal mean blur rate (MBR) (Nasal) was significantly lower in multivessel disease than one-vessel disease group (6.6 ± 1.6 vs. 8.5 ± 2.6, *P* = 0.006). This was not significant for retinal MBR or choroidal MBR (Temporal).

**Fig. 2:**
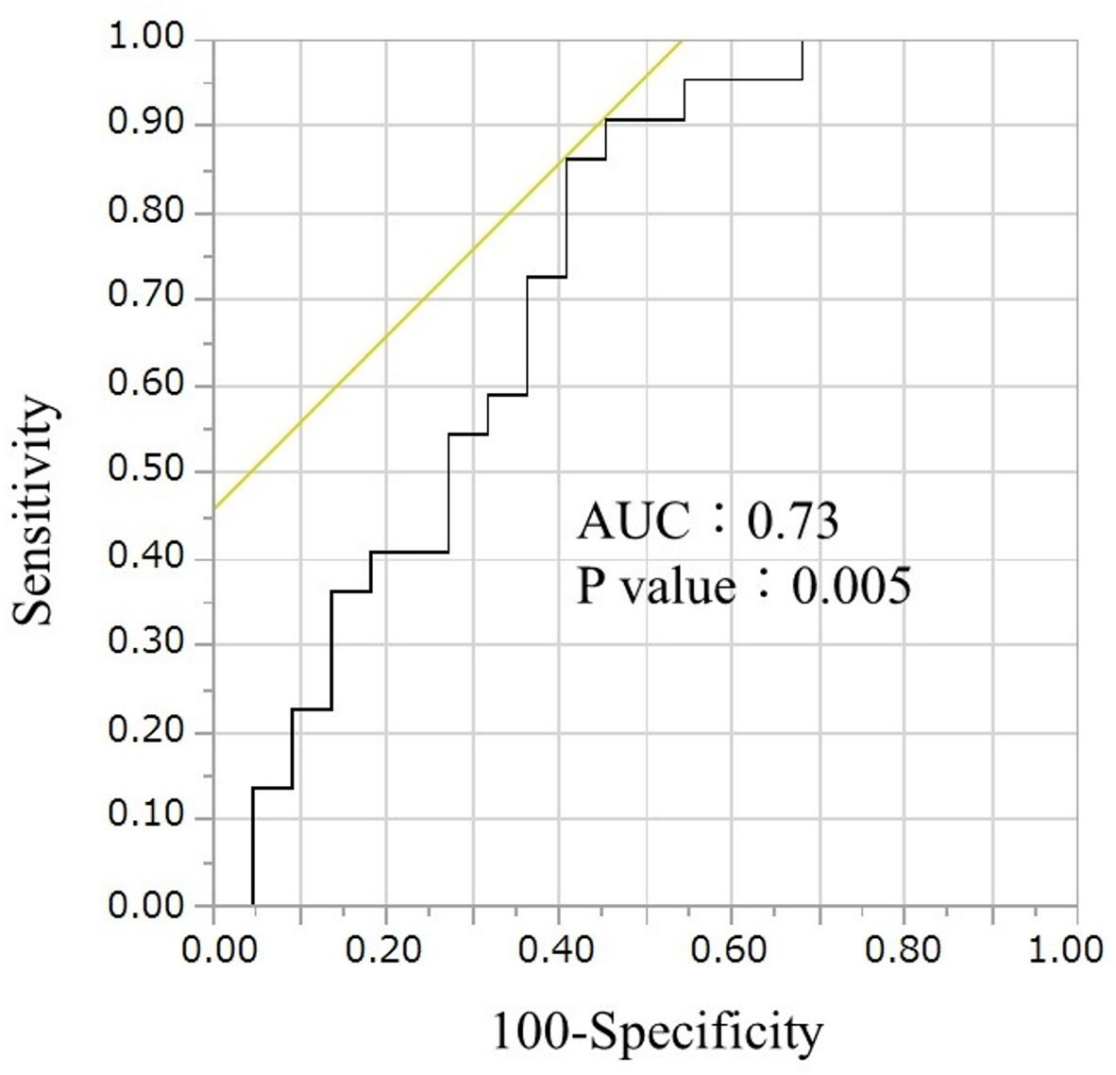
The receiver operating characteristics (ROC) curve of the choroidal mean blur rate (MBR) predicts multivessel disease. ROC analysis revealed that the cut-off value of choroidal MBR (Nasal) to predict patients with multivessel disease was 8.7 (area under ROC curve: 0.73, sensitivity: 0.91, specificity: 0.55, *P* = 0.005).

The choroidal MBR (nasal) was significantly lower in the multivessel disease group than that in the one-vessel disease group (6.6 ± 1.6 vs. 8.5 ± 2.6, *P* = 0.006) (Fig. 1); however, the retinal or choroidal MBR (temporal) were not significantly different between groups. The receiver operating characteristics (ROC) curve of choroidal MBR for predicting multivessel disease is shown in Fig. 2. The ROC analysis revealed that the cut-off value of choroidal MBR (nasal) to identify patients with multivessel disease was 8.7 (area under ROC curve: 0.73, sensitivity: 0.91, specificity: 0.55, *P* = 0.005).

### Relationship of systemic atherosclerosis findings to retinal and choroidal MBR

To explore potential associations, we first examined the simple correlations between systemic atherosclerosis findings and retinal and choroidal MBR. While multivariate analyses including covariates were also performed to account for confounding factors, statistically significant relationships were not observed, likely owing to the limited sample size (*n* = 44) of this exploratory study.

Table 2 shows the relationship between systemic atherosclerosis and the retinal and choroidal MBR. Retinal MBR was negatively correlated with the Left ventricular (LV).

**Table 2:** Relationship between the retinal MBR and choroidal MBR and systemic atherosclerosis parameters.

Variables	Retinal MBR		Choroidal MBR(Temporal)		Choroidal MBR(Nasal)	
	r	P value	r	P value	r	P value
A. **Systemic parameters**						
IMT	-0.30	0.051	0.027	0.87	-0.30	0.058
baPWV	-0.13	0.41	0.26	0.087	**0.33**	**0.029**
LDL-Cho	0.017	0.91	0.065	0.68	0.96	0.54
TG	0.074	0.63	-0.048	0.76	0.0006	1.0
HbA1c	-0.069	0.66	0.19	0.21	0.047	0.76
B. **Renal parameters**						
BUN	-0.28	0.070	0.063	0.69	-0.089	0.56
Creatinine	-0.24	0.12	-0.16	0.29	-0.11	0.47
eGFR	0.12	0.44	0.11	0.49	0.12	0.42
C. **Cardiac parameters**						
SYNTAX score	-0.28	0.063	0.016	0.92	0.026	0.87
LVDd	-0.19	0.20	**-0.30**	**0.045**	-0.15	0.34
LVDs	-0.26	0.086	-0.22	0.15	-0.19	0.21
LVEF	0.22	0.15	0.12	0.42	-0.008	0.96
LVmass index	**-0.36**	**0.02**	-0.18	0.24	-0.09	0.57
E/e’	-0.30	0.047	-0.043	0.78	-0.11	0.48

**Fig. 3:**
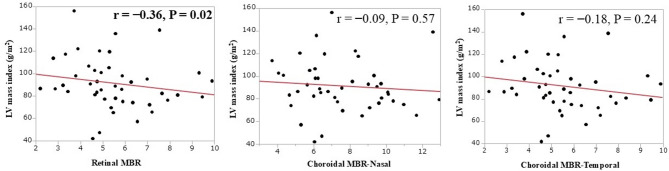
The retinal mean blur rate (MBR) was negatively correlated with left ventricular (LV) mass index (*r* = − 0.36, *P* = 0.02). This was not significant for choroidal MBR (Nasal) or choroidal MBR (Temporal).

**Fig. 4:**
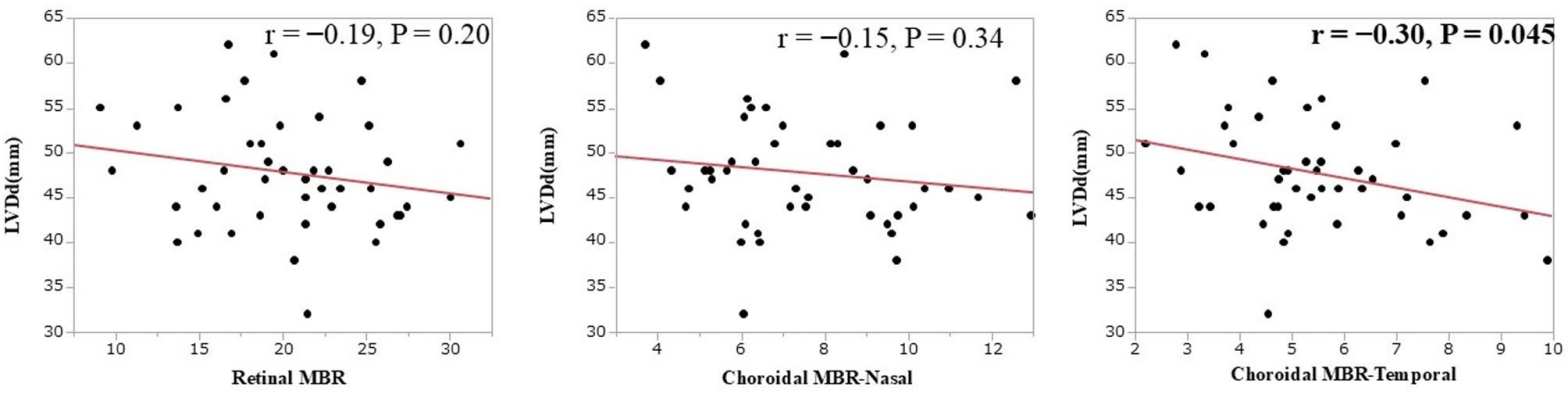
The choroidal mean blur rate (MBR) (Temporal) was negatively correlated with LVDd (*r* = − 0.30, *P* = 0.045). This was not significant for retinal MBR or choroidal MBR (Nasal).

**Fig. 5:**
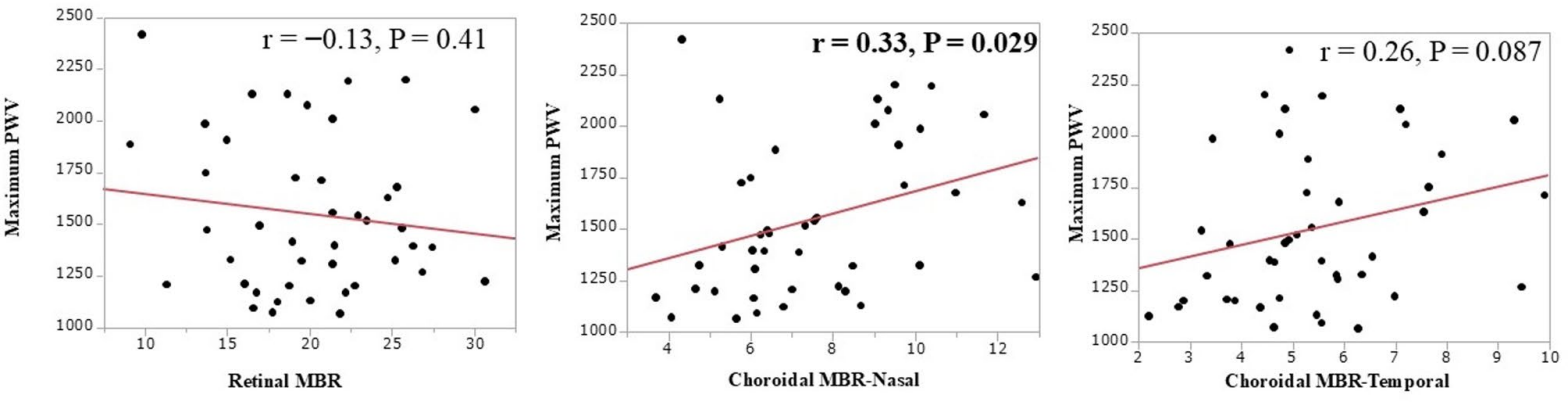
The choroidal mean blur rate (MBR) (Nasal) was positively correlated with baPWV (*r* = 0.33, *P* = 0.029). This was not significant for retinal MBR or choroidal MBR (Temporal).

Abbreviations are shown in Table 1.

mass index (*r* = − 0.36, *P* = 0.02) (Fig. 3). The choroidal MBR (temporal) was negatively correlated with left ventricular diastolic diameter (LVDd) (*r* = − 0.30, *P* = 0.045) (Fig. 4), and the choroidal MBR (nasal) was positively correlated with baPWV (*r* = 0.33, *P* = 0.029) (Fig. 5).

## Discussion

This study has three main findings. First, nasal choroidal MBR is useful in determining the presence of MVD in patients with ACS. Second, choroidal MBR was associated with findings of systemic arteriosclerosis. Finally, choroidal MBR in the nasal and temporal regions was associated with various systemic parameters, including the coronary arteries, in aspects different from those of retinal MBR.

### Clinical value of choroidal MBR measured by LSFG

Choroidal blood flow is the primary source of oxygen and nutrients for the choroid and outer retina. Reduced choroidal blood flow is associated with several ocular diseases including glaucoma, retinitis pigmentosa, and age-related macular degeneration^11^. The LSFG can provide quantitative estimates of ocular blood flow, including choroidal blood flow^4^. Notably, consistent with our findings using LSFG, previous studies employing different ophthalmic modalities have also reported reduced choroidal blood flow in patients with ACS^12^. These independent observations further underscore the clinical relevance of assessing choroidal circulation in this patient population and reinforce our conclusion that choroidal circulatory impairment is a feature in ACS patients. The rate of the speckle pattern change in the LSFG was expressed numerically, and the flow rate was calculated as the MBR. Although the LSFG index is the only method that can directly and non-invasively evaluate arteriosclerosis of the microvessels, it is unclear whether the choroidal blood flow index calculated by the LSFG is due to ocular or systemic atherosclerosis, or both. This study addresses this question and presents several important findings.

MVD is associated with more complications and poorer in-hospital outcomes than those of single-vessel diseases. In addition, MVD is more frequently associated with older age, diabetes, and hypertension than single-vessel disease^13^, and the CVI, which is measured in the choroid, is useful in identifying MVD^8^. It is difficult to assess choroidal CVI for the presence of CAD, but it can be useful in differentiating mild from severe CAD. In this study, age and a history of diabetes or hypertension were not associated with the presence or absence of MVD. Although this study was conducted in patients with ACS, choroidal blood flow measured using LSFG was associated with the presence of MVD, suggesting that choroidal blood flow impairment may be useful in distinguishing MVD from severe CAD, as in previous studies.

Carotid artery assessment using ultrasonography effectively detects the presence of carotid and other atherosclerotic diseases^14^. High mean intima-media thickness (IMT) is a major cardiovascular risk factor^15^, and baPWV, the most widely used measure of arterial stiffness, is a strong predictor of future cardiovascular events, such as ACS^16^. The cardio-ankle vascular index (CAVI) was correlated with the CVI in diabetic groups, and systemic sclerosis was associated with choroidal blood flow impairment^17^. Retinal blood flow impairment detected by LSFG is associated with baPWV and higher IMT^5^, but the association between choroidal blood flow impairment, as measured by the LSFG, and baPWV or IMT has not been previously reported. In this study, choroidal blood flow measured by LSFG was significantly correlated with baPWV but not with IMT. IMT measures intima-media thickening due to plaque accumulation in the arteries, whereas baPWV measures the presence of an increased pulse wave velocity of blood flow associated with arterial stiffness. In other words, IMT assesses the progression of atherosclerosis, whereas baPWV does not necessarily reflect atherosclerosis. Therefore, we believe that the results obtained in this study showing that baPWV, which mainly reflects arteriosclerosis, is associated with ocular blood flow impairment, which is mainly caused by arteriosclerosis, are reasonable. This suggests that choroidal microcirculation may be more closely associated with the functional aspect of systemic arteriosclerosis (arterial stiffness) rather than the structural aspect (atherosclerotic plaque burden). This study suggests that systemic arteriosclerosis may impair retinal blood flow, making it necessary to evaluate both ocular and systemic arteriosclerosis.

The nasal side of the fundus has abundant blood flow because it contains the optic nerve head and is on the central side of the ocular vasculature, whereas the temporal side is on the peripheral side of the ocular vasculature and is considered to have less blood flow than that on the nasal side. In this study, the choroidal MBR (nasal) showed correlations with the presence of coronary artery multivessel disease and baPWV, a systemic arteriosclerosis index. However, the fact that these correlations were observed on the nasal side, where blood flow is abundant, was convincing in terms of blood flow dynamics. In addition, the finding that choroidal MBR (temporal), an index of peripheral ocular blood flow, correlated with LVDd, an index of left ventricular remodeling, is similar to that of a previous report describing the effect of microvascular occlusion on left ventricular remodeling^18^. This specific association in the temporal region may reflect physiological differences in ocular vascular architecture. The nasal choroid, being closer to the optic nerve head, typically contains larger, more central vessels and may exhibit less variability in blood flow. Conversely, the temporal choroid represents a more peripheral microvascular bed, which could be more susceptible to systemic hemodynamic influences, particularly those arising from changes in cardiac diastolic function or left ventricular remodeling. As left ventricular remodeling and diastolic dysfunction are known to affect systemic peripheral vascular resistance and microcirculatory blood flow, these effects are plausibly reflected in the more vulnerable peripheral choroidal blood flow of the temporal region. Furthermore, retinal blood flow assessment measured by LSFG correlates with LV mass, and the fact that retinal MBR correlated with the LV mass index in the present study is consistent with the findings of previous reports^19^. An increased LV mass is associated with hypertension and is an indicator of pressure overload in the heart^20^. Retinal arterioles are not innervated by the autonomic nervous system and have a self-regulatory mechanism for blood flow, which may make them less susceptible to blood pressure fluctuations. However, they may also be more susceptible to sustained hypertension than choroidal arterioles^21^. While we investigated various echocardiographic parameters, including left ventricular systolic diameter (LVDs), left ventricular ejection fraction (EF), and E/e’ as an indicator of diastolic function, these did not show significant correlations with choroidal MBR in our study. This suggests that choroidal and retinal microcirculation might be more sensitive to chronic structural cardiac remodeling (e.g., LVDd, LV mass index) and long-term diastolic loading conditions^22^, rather than overall reductions in cardiac systolic function or more acute changes in filling pressures. The long-term cardiac structural changes reflected by LVDd and LV mass index may manifest as more chronic effects on the vascular bed, aligning with previous research indicating a link between systemic microcirculatory changes and cardiac structural and functional alterations^23^.

This study suggests that systemic arteriosclerosis may impair retinal blood flow, making it necessary to evaluate both ocular and systemic arteriosclerosis. However, it is important to note that while we observed significant simple correlations, the limited sample size of this preliminary study prevented us from establishing statistically significant adjusted relationships in multivariate analyses. Future larger-scale investigations are warranted to further explore and confirm these complex associations, accounting for potential confounding variables.

### Clinical implications

Our results indicate the clinical importance of the non-invasive evaluation of choroidal blood flow as a robust marker for promoting comprehensive evaluations of not only choroidal but also systemic arteriosclerosis in patients with ACS. In addition, its usefulness in identifying MVD in patients with ACS suggests that choroidal blood flow may be a predictor of cardiovascular disease progression.

## Study limitations

This study had several limitations. First, this study was conducted at a single institution. The sample size was small because we focused on patients with ACS not requiring hemodialysis or an assisted circulation apparatus. Second, because most of the patients with ACS were men, sex differences may have influenced the results^24^. Third, the timing of our comprehensive evaluation, which included ophthalmic and other modality assessments, may have influenced the results due to the relatively acute phase of ACS. We attempted to minimize this potential impact by implementing strict exclusion criteria, such as excluding patients requiring hemodialysis or mechanical circulatory support. Furthermore, all assessments were performed in patients who were considered to have a stable physical condition within a one-week period following the initial blanking period after ACS onset. This approach was intended to ensure that the evaluations were conducted at a consistent and medically stable time point for all participants. Finally, while we acknowledge the importance of adjusting for covariates, our multivariate analyses did not yield statistically significant results when including these factors, likely due to the relatively small sample size (*n* = 44). This is a known challenge in exploratory studies with limited participant numbers and represents a limitation of our current findings, suggesting a need for larger studies to confirm these relationships. Consequently, the significant univariate findings, while clinically suggestive, should be considered exploratory and warrant further validation in future studies.

## Conclusion

This study demonstrates that choroidal blood flow, as evaluated by LSFG in patients with ACS, is specifically associated with functional systemic arteriosclerosis (arterial stiffness) and left ventricular diastolic remodeling (LVDd). Furthermore, we found significant differences in choroidal blood flow between patients with and without severe coronary artery disease (multivessel disease). These findings suggest that choroidal MBR could serve as a valuable non-invasive marker for a comprehensive assessment of systemic vascular health, particularly reflecting arterial stiffness, chronic cardiac structural changes, and the severity of coronary artery disease in ACS patients.

## Methods

Fifty-eight patients admitted to the coronary care unit of Nihon University Itabashi Hospital for ACS between April 1, 2019, and September 30, 2020, were studied. All studied patients underwent coronary artery angiography via the femoral/radial approach with a 6-Fr catheter. All coronary segments were interpreted visually by two or more experienced cardiologists. We defined significant stenosis on coronary angiography as > 50% stenosis of an epicardial coronary artery^25^. We investigated whether multivessel and left main coronary lesions were involved in assessing the extent and severity of the CAD cases. All patients underwent PCI for culprit lesion on admission, followed by a visit to the Department of Ophthalmology (mean, 11 ± 5 days after ACS) once their general condition stabilized. The dates for comprehensive assessment by LSFG, ultrasonographic imaging of the carotid artery, baPWV, and transthoracic echocardiography were set within 1 week between the four modalities. Patients were excluded from the study if they had glaucoma, uveitis, optic neuropathy, retinal or choroidal vascular disease, or a LV ejection fraction < 30%, and if they underwent hemodialysis, had previous intraocular surgery, used an assisted circulation apparatus, or if they were unable to visit the Department of Ophthalmology on foot. Finally, 44 patients met the study criteria. The Institutional Review Board of Nihon University Itabashi Hospital approved this cross-sectional study, and all participants provided informed consent for participation in the study. This study was conducted in accordance with the tenets of the Declaration of Helsinki.

### Measurement of the carotid intima-media thickness

One to two weeks after admission for ACS, high-resolution ultrasonographic imaging of the carotid artery was performed with an EUB-8500 device (Hitachi, Co. Ltd., Tokyo, Japan) using the B-scan mode and a probe frequency of 7.5 MHz. Measurements were performed with the participants in the supine position and their heads slightly turned away from the sonographer. The procedures involved scanning the near and far walls of the carotid artery 1 cm proximal and distal to the carotid bulb in longitudinal view, and the average of the maximum values on both sides was used as the mean IMT for the data analysis^26^.

### Measurements of brachial-ankle pulse-wave velocity

The baPWV was measured 1–2 weeks after admission for ACS using a volume-plethysmographic device (baPWV/ABI; Nihon Colin Co., Tokyo, Japan) that simultaneously recorded heart sounds, electrocardiograms, and blood pressures in the left and right brachia and ankles. After the patients rested for a minimum of 5 min in the supine position, pulse volume waveforms were recorded noninvasively over the brachial and tibial arteries, and the time delay (T) between the two-waveform feet was measured. The distance (D) covered by the waves was estimated as the distance between the two recording sites. The baPWV was calculated as follows: baPWV = D (cm)/T(s).

### Laboratory measurements

The following values were measured during transport to our emergency department: blood urea nitrogen (mg/dL), creatinine (mg/dL), estimated glomerular filtration rate (mL/min/1.73 m2), low-density lipoprotein cholesterol (mg/dL), triglycerides (mg/dL), and HbA1c (%).

### Echocardiographic parameters

Echocardiographic parameters were obtained during the stable phase (mean, 6 ± 4 days after admission for ACS). Echocardiography was performed with the patient in the supine position using Vivid 7 or Vivid E9 cardiovascular ultrasonographic systems (GE Healthcare, Milwaukee, WI, USA) operated by experienced sonographers who were blinded to patient data. Echocardiographic measurements were performed in accordance with the American Society of Echocardiography guideline^27^. Briefly, the LVDd, LV systolic diameter (LVDs), and left atrial diameter were measured in the parasternal long-axis view. The LV ejection fraction was measured using the modified Simpson method in apical four- and 2-chamber views. LV mass was calculated using a formula derived from the American Society of Echocardiography^28^. The LV mass index was calculated as the ratio of LV mass to body surface area. Transmitral flow velocity curves were recorded to measure peak early (E) and late (A) diastolic velocities. Tissue Doppler imaging at the mitral annulus level was performed in the septal position to measure the early (e’) and late (A’) diastolic myocardial velocities^29^.

### Ocular fundus examinations

All patients underwent baseline ophthalmic evaluation by a well-trained ophthalmologist (HY) before ocular blood flow measurement. All patients had good visual acuity (VA > 20/20) and normal intraocular pressure (IOP < 20 mm Hg). The IOP was monitored using applanation tonometry (Haag Streit, Bern, Switzerland).

### Laser speckle flowgraphy measurements

**Fig. 6:**
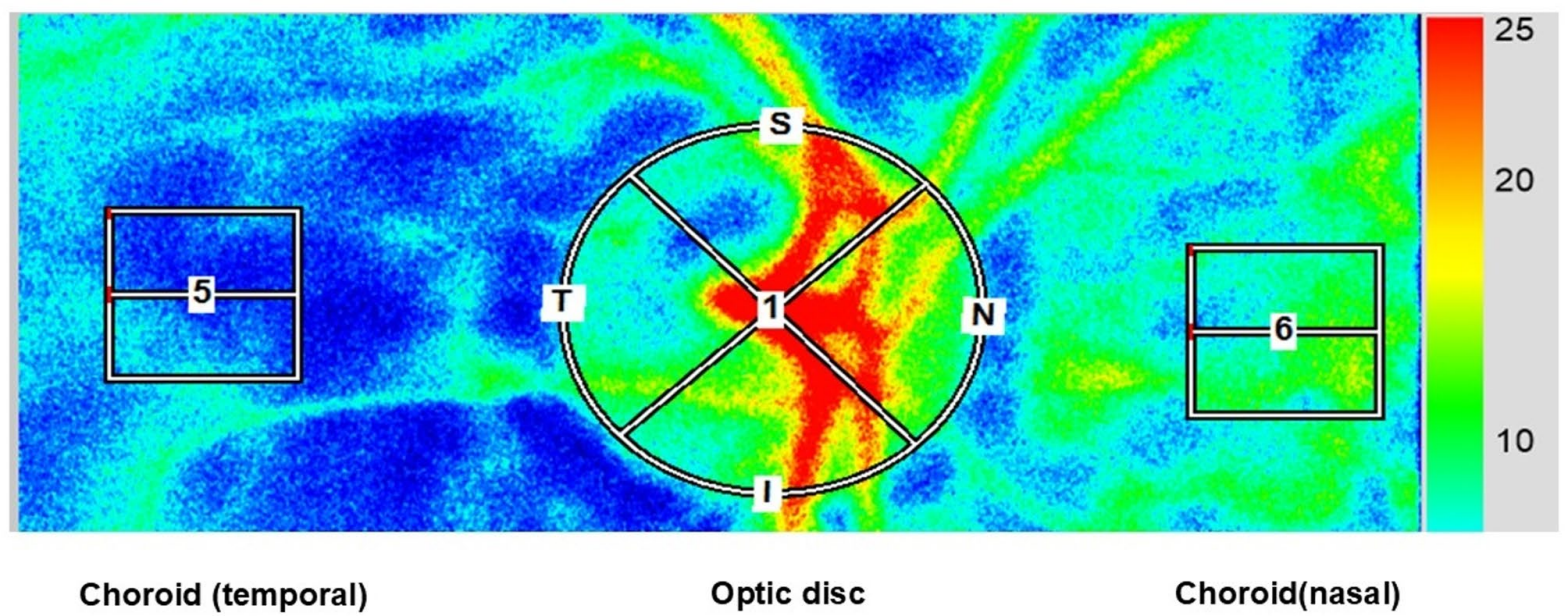
Methods of determining the pulse-wave velocity in the optic nerve head and choroidal circulation by laser speckle flowgraphy. This is a circle defining the area for measurements in three regions, optic disc and choroidal region 1 disc diameter from the disc choroid temporally and nasally.

After pupils were dilated with 0.5% tropicamide eye drops, a commercially available LSFG-NAVI system (Softcare Co., Ltd., Fukutsu, Japan) was used to measure ocular circulation in the optic nerve head (ONH). The principles of the LSFG have been previously described in detail^30^. Briefly, LSFG images were obtained from a 21° section centered on the optic disc. This observation field was sized 750 pixels (width) × 360 pixels (height). The MBR was calculated from moving erythrocytes illuminated by an 830-nm wavelength diode laser beam. Mean blur rates were expressed in arbitrary units and considered an indicator of relative erythrocyte velocity. A total of 118 MBR images were recorded from the ONH area over 4 s. Using an accompanying analysis software (LSFG Analyzer, Version 3.3.3.0; Softcare Co., Ltd., Fukutsu, Japan)^31^, a colorscale map of the still images was automatically created by averaging the MBR images (Fig. 6). In this study, we measured the MBR at three locations in each eye, the ONH region, and one disc diameter from the ONH region to the temporal and nasal choroidal regions, respectively, twice for each eye; the average values calculated by the LSFG software were used for the statistical analysis. We placed rubber bands in the form of 100 × 100 rectangles on both the temporal and nasal sides of the choroid. The nasal side exhibits a denser vascular structure compared to the temporal side. In contrast, the temporal side is typically located within the watershed area, which is characterized by reduced blood flow. In total, we set two rubber bands—one on each side of the choroid—each representing different blood flow characteristics.

### Statistical analyses

Continuous data are expressed as mean ± standard deviation if normally distributed, or as median (interquartile range), if otherwise. Comparisons were performed using Student t-test or Mann–Whitney U test. Categorical data are expressed as numbers and percentages and were compared using the chi-squared test or Fisher exact test. Linear regression analysis with Spearman rank-order correlation coefficients was used to assess the correlations between the variables, including systemic atherosclerosis, renal and transthoracic echocardiography cardiac parameters, and the LSFG parameters. Statistical significance was defined as a 2-tailed P-value < 0.05. Statistical analyses were performed using JMP Version 14.0 (SAS Institute, Cary, NC, USA).

## Data Availability

The authors confirm that the data supporting the findings of this study are available in the article and supplementary material.

## Abbreviations

A: peak mitral A wave velocity
AMI: acute myocardial infarction
ARB: angiotensin receptor blocker
baPWV: brachial-ankle pulse-wave velocity
BUN: blood urea nitrogen
CKD: chronic kidney disease
CVI: Choroidal Vascularity Index
E: peak mitral E wave velocity
e’: peak early diastolic myocardial velocity at septal position recorded by tissue Doppler imaging
E/e’: ratio, ratio of peak mitral E wave velocity to peak early diastolic myocardial velocity at the septal position by tissue Doppler imaging
eGFR: estimated glomerular filtration rate
HbA1c: hemoglobin A1c
HDL-Cho: high-density lipoprotein cholesterol
IMT: intima-media thickness
IOP: intraocular pressure
LDL-Cho: low-density lipoprotein cholesterol
LSFG: laser speckle flowgraphy
LV: left ventricular
MBR: mean blur rate
MVD: multivessel disease
ONH: optic nerve head
PCI: percutaneous coronary intervention
